# Adults with *RRM2B*-related mitochondrial disease have distinct clinical and molecular characteristics

**DOI:** 10.1093/brain/aws231

**Published:** 2012-10-29

**Authors:** Robert D. S. Pitceathly, Conrad Smith, Carl Fratter, Charlotte L. Alston, Langping He, Kate Craig, Emma L. Blakely, Julie C. Evans, John Taylor, Zarfishan Shabbir, Marcus Deschauer, Ute Pohl, Mark E. Roberts, Matthew C. Jackson, Christopher A. Halfpenny, Peter D. Turnpenny, Peter W. Lunt, Michael G. Hanna, Andrew M. Schaefer, Robert McFarland, Rita Horvath, Patrick F. Chinnery, Douglass M. Turnbull, Joanna Poulton, Robert W. Taylor, Gráinne S. Gorman

**Affiliations:** 1 MRC Centre for Neuromuscular Diseases, UCL Institute of Neurology and National Hospital for Neurology and Neurosurgery, Queen Square, London, WC1N 3BG, UK; 2 Oxford Medical Genetics Laboratories, Churchill Hospital, Oxford, OX3 7LE, UK; 3 Mitochondrial Research Group, Newcastle University, Newcastle upon Tyne, NE2 4HH, UK; 4 Department of Neurology, Universität Halle-Wittenberg, 06097 Halle, Germany; 5 Department of Cellular Pathology, Queen’s Hospital, Romford, RM7 0AG, UK; 6 Department of Neurology, Salford Royal NHS Foundation Trust, Salford, M6 8HD, UK; 7 Department of Neurology Wycombe General Hospital, High Wycombe, HP11 2TT, UK; 8 Department of Neurology, Queen Alexandra Hospital, Portsmouth, PO6 3LY, UK; 9 Department of Clinical Genetics, Royal Devon and Exeter Hospital, Exeter, EX2 5DW, UK; 10 Department of Clinical Genetics, Gloucester Royal Hospital, Gloucester, GL1 3NN, UK; 11 Institute of Genetic Medicine, Newcastle University, Newcastle upon Tyne, NE1 3BZ, UK; 12 Nuffield Department of Obstetrics and Gynaecology, University of Oxford, Oxford, OX3 9DU, UK

**Keywords:** mitochondrial DNA, mtDNA maintenance, mtDNA depletion, multiple mitochondrial DNA deletions, *RRM2B*

## Abstract

Mutations in the nuclear-encoded mitochondrial maintenance gene *RRM2B* are an important cause of familial mitochondrial disease in both adults and children and represent the third most common cause of multiple mitochondrial DNA deletions in adults, following *POLG* [polymerase (DNA directed), gamma] and *PEO1* (now called *C10ORF2*, encoding the Twinkle helicase) mutations. However, the clinico-pathological and molecular features of adults with *RRM2B*-related disease have not been clearly defined. In this multicentre study of 26 adult patients from 22 independent families, including five additional cases published in the literature, we show that extra-ocular neurological complications are common in adults with genetically confirmed *RRM2B* mutations. We also demonstrate a clear correlation between the clinical phenotype and the underlying genetic defect. Myopathy was a prominent manifestation, followed by bulbar dysfunction and fatigue. Sensorineural hearing loss and gastrointestinal disturbance were also important findings. Severe multisystem neurological disease was associated with recessively inherited compound heterozygous mutations with a mean age of disease onset at 7 years. Dominantly inherited heterozygous mutations were associated with a milder predominantly myopathic phenotype with a later mean age of disease onset at 46 years. Skeletal muscle biopsies revealed subsarcolemmal accumulation of mitochondria and/or cytochrome *c* oxidase-deficient fibres. Multiple mitochondrial DNA deletions were universally present in patients who underwent a muscle biopsy. We identified 18 different heterozygous *RRM2B* mutations within our cohort of patients, including five novel mutations that have not previously been reported. Despite marked clinical overlap between the mitochondrial maintenance genes, key clinical features such as bulbar dysfunction, hearing loss and gastrointestinal disturbance should help prioritize genetic testing towards *RRM2B* analysis, and sequencing of the gene may preclude performance of a muscle biopsy.

## Introduction

Approximately one-half of all adult mitochondrial diseases result from genetic dysfunction of the nuclear-encoded mitochondrial genes. A significant number of these cases are due to disruption of genes involved in maintenance (replication and repair) of mitochondrial DNA, leading to qualitative (accumulation of multiple mitochondrial DNA deletions) and/or quantitative (depletion of mitochondrial DNA copy number) downstream mitochondrial genomic effects. To date, almost all known nuclear maintenance genes fall into one of two categories: (i) genes whose protein products function directly at the mitochondrial DNA replication fork (Van Goethem *et al.*, 2001; Spelbrink *et al.*, 2001; Longley *et al.*, 2006); or (ii) genes that encode proteins involved in supplying mitochondria with deoxyribonucleoside triphosphate (dNTP) pools required for DNA replication (Nishino *et al.*, 1999; Kaukonen *et al.*, 2000; Mandel *et al.*, 2001; Saada *et al.*, 2001; Bourdon *et al.*, 2007; Ostergaard *et al.*, 2007; Fratter *et al.*, 2011). The most common presenting neurological feature seen in adults with mitochondrial DNA maintenance disorders is progressive external ophthalmoplegia (PEO) and ptosis. Of the 12 known maintenance genes, eight have been associated with PEO, although the clinical phenotype is not necessarily restricted to the extra-ocular muscles.

Ribonucleotide reductase M2B (TP53 inducible), *RRM2B* (MIM 604 712), encodes the p53-inducible small subunit (p53R2) of ribonucleotide reductase, a heterotetrameric enzyme that catalyses *de novo* syntheses of dNTPs by direct reduction of ribonucleoside diphosphates to their corresponding deoxyribonucleoside diphosphates. This process supplements the dNTPs produced by the mitochondrion’s own dNTP salvage pathway, defects of which cause many of the mitochondrial DNA depletion syndromes (Rahman and Poulton, 2009), and is essential for mitochondrial DNA synthesis. Transcription of *RRM2B* is tightly regulated by the tumour suppressor protein p53. Mutations in the gene have been associated with both recessively and dominantly inherited mitochondrial disease. Spliced variant transcripts have also been reported (Spinazzola *et al.*, 2009).

The first reported human diseases linked to *RRM2B* dysfunction were associated with mitochondrial DNA depletion (Bourdon *et al.*, 2007; Bornstein *et al.*, 2008; Acham-Roschitz *et al.*, 2009; Kolberg *et al.*, 2009; Spinazzola *et al.*, 2009) and caused severe, early-onset, multisystem disease with infant mortality. *RRM2B* mutations have since been recognized to cause adult-onset syndromes. This was first demonstrated in a patient with mitochondrial neurogastrointestinal encephalopathy and mitochondrial DNA depletion (Shaibani *et al.*, 2009). Tyynismaa *et al.* (2009) later published about a large autosomal-dominant PEO family with multiple mitochondrial DNA deletions whose disease was not explained by mutations in any of the known autosomal-dominant PEO genes. The disease locus in the family was mapped using genome-wide linkage and found to contain *RRM2B*, and a heterozygous nonsense mutation in exon 9 was detected and demonstrated in another unrelated family. The mutant messenger RNA was shown to escape nonsense-mediated decay and resulted in a truncated protein that was postulated to cause a dominant-negative or gain-of-function effect on the heterotetrameric structure of the ribonucleotide reductase enzyme. The mechanistic importance of truncating exon 9 mutations was further expanded by Fratter *et al.* (2011), who showed that three further novel pathogenic variants in exon 9 caused familial autosomal-dominant PEO in seven unrelated probands. In addition, clinically more severe, recessively inherited, compound heterozygous mutations were demonstrated. Kearns–Sayre syndrome resulting from compound heterozygous *RRM2B* mutations with secondary multiple mitochondrial DNA deletions has also been reported, thus confirming the more severe nature of recessively inherited adult *RRM2B* mutations and expanding the clinical phenotype associated with *RRM2B*-related mitochondrial disease beyond that of PEO and PEO-plus syndromes (Pitceathly *et al.*, 2011).

Despite emerging evidence that *RRM2B* mutations are the third most common cause of multiple mitochondrial DNA deletions in adults, following *POLG* and *PEO1* (now known as *C10ORF2*) (Fratter *et al.*, 2011; Pitceathly *et al.*, 2011), and *RRM2B* dysfunction being an important cause of neurological disease, the adult phenotypic and molecular spectrum is not fully understood. We, therefore, conducted a systematic clinical and molecular study of adult patients with *RRM2B* mutation(s) to define the phenotypic spectrum of adult *RRM2B*-related mitochondrial disease and establish any genotype–phenotype correlations.

## Materials and methods

### Patient cohorts

#### Newcastle–Oxford–Halle cohort

We selected a cohort of adult patients who had been referred to the NHS Specialized Services-funded Mitochondrial Diagnostic Centres in Newcastle and Oxford, UK, or the Mitochondrial Centre in Halle, Germany, for investigation of suspected mitochondrial disease, owing to their clinical presentation, evidence of a mitochondrial biochemical defect and/or presence of mitochondrial DNA deletions. *RRM2B* sequencing was performed when initial screens for *POLG*, *POLG2*, *PEO1* and *SLC25A4* were found to be negative.

The subject’s consent was obtained according to the Declaration of Helsinki, and institutional ethical committee approval was obtained at each centre in which the work was performed.

#### Additional published adult cases

We conducted a systematic review of the literature to identify all previously published adult cases with confirmed *RRM2B* mutations. These were included in our analysis to determine the complete clinical spectrum and genotype–phenotype correlates seen in adult *RRM2B*-related mitochondrial disease (Shaibani *et al.*, 2009; Tyynismaa *et al.*, 2009; Fratter *et al.*, 2011; Pitceathly *et al.*, 2011; Takata *et al.*, 2011).

### Phenotypic evaluation

To define the extent and severity of patient’s mitochondrial disease, a neurologist at each centre comprehensively assessed each patient harbouring pathogenic *RRM2B* mutations and/or examined their medical records.

### Mitochondrial histochemistry

Skeletal muscle biopsies were collected from 21 patients with pathogenic *RRM2B* mutations. Cryostat sections (10 µm) were cut from transversely oriented muscle blocks and subjected to cytochrome *c* oxidase (COX), succinate dehydrogenase (SDH) and sequential COX-SDH histochemical staining to assess the numbers of COX-deficient fibres as a measure of respiratory chain deficiency, as previously described (Taylor *et al.*, 2004). The SDH reaction was used to ascertain the number of fibres exhibiting increased levels of enzyme activity in the subsarcolemmal region, namely, ‘ragged-blue’ fibres, in addition to data obtained from the histological modified Gomori trichrome stain.

### Mitochondrial DNA analysis

Where available, total muscle DNA was extracted by standard techniques and screened for mitochondrial DNA rearrangements by Southern blotting and/or long-range PCR protocols, as described previously (Murphy *et al.*, 2008). The presence of clonally expanded mitochondrial DNA deletions in individual COX-positive and COX-deficient fibres was further screened in muscle biopsies from nine patients with either dominantly inherited or recessively inherited *RRM2B* mutations (Patients 1.1, 3, 5, 9, 10, 14, 19 and 20), by previously published protocols, using primers and TaqMan® probes to assess the simultaneous amplification of the *MTND1* (rarely deleted) and *MT-ND4* (often deleted) genes (He *et al.*, 2002; Krishnan *et al.*, 2007). Absence of mitochondrial DNA depletion in muscle was confirmed in all cases by real-time quantitative PCR, as described previously (Blakeley *et al.*, 2008).

### Identification of pathogenic *RRM2B* mutations

The coding exons and intron–exon boundaries of *RRM2B* (GenBank accession number NM_015713) were sequenced, as previously described (Tyynismaa *et al.*, 2009). *RRM2B* exon copy number (exons 1–8) was assessed by multiplex ligation-dependent probe amplification (MRC-Holland kit P089-A1) in all patients with dominantly inherited heterozygous missense *RRM2B* mutations. Total RNA from patients with the c.48G>A *RRM2B* variant (Patients 1, 1.1 and 2) was extracted from Ethylenediaminetetraacetic acid-blood with the QIAamp® RNA Blood Mini kit (Qiagen) and reverse transcribed using the ThermoScript™ RT-PCR system (Invitrogen). The resulting complementary DNA was PCR-amplified across the *RRM2B* exon 1–2 boundary using exonic primers, leading to a normally spliced 303-bp product. The PCR products were separated by agarose gel electrophoresis under standard conditions. Gel stabs of discreet bands were taken for secondary PCR using the same primers, which were M13-tagged for subsequent dideoxy Sanger sequencing using universal M13 primers, BigDye® Terminator kit 3.1 (Applied Biosystems) and capillary electrophoresis on an ABI Prism 3730 Genetic Analyser.

### Modelling missense *RRM2B* mutations on the p53R2 crystal structure

The 2.6 Å X-ray crystal structure of human p53R2 (Research Collaboratory for Structural Bioinformatics Protein Data Bank: reference 3HF1) (Smith *et al.*, 2009) was viewed in ViewerLite v.4.2 (Accelrys Inc.), and the amino acids known to have been substituted were isolated and saved as a separate pdb file. The p53R2 crystal structure and the isolated amino acids were then rendered using PyMOL v.0.97 (Warren L. DeLano ‘The PyMOL Molecular Graphics System’ DeLano Scientific LLC, http://www.pymol.org).

## Results

### Patient cohorts

Clinical data are presented on 31 patients (Table 1). Twenty-six patients were identified by the NHS Specialized Services-funded Mitochondrial Diagnostic Centres in Newcastle and Oxford, UK, or the Mitochondrial Centre in Halle, Germany (Newcastle–Oxford–Halle cohort), and are described here in greater clinical and molecular detail. Clinical information from five further patients was obtained from the literature review. One previously published case (Patient 12, Fratter *et al.*, 2011) was omitted from the current analysis, as the pathogenic nature of the reported heterozygous mutation was uncertain after segregation studies.

**Table 1:** Summary of clinical data from 31 patients with pathogenic *RRM2B* mutations

Clinical presentation	Number of patients	Per cent of all patients
Ophthalmoparesis	31	100
Ptosis	28	90
Proximal muscle weakness	16	52
Bulbar dysfunction	13	42
Ataxia	12	39
Fatigue	11	36
Sensorineural hearing loss	11	36
Endocrinopathy	7	23
Gastrointestinal disturbance	6	19
Cognitive impairment/learning difficulties	4	13
Encephalopathy/stroke-like episode	3	10
Renal disturbance	3	10
Depression	3	10
Neuropathy	2	6
Pigmented retinopathy	2	6
Cardiac dysfunction/arrhythmia	2	6
Cataracts	2	6
Migraine	2	6
Distal muscle weakness	1	3
Glaucoma	1	3
Short stature	1	3

#### Newcastle–Oxford–Halle cohort

The clinical and molecular features of all 26 patients from our Newcastle–Oxford–Halle cohort are summarized in Table 2. Molecular data from 11 of these subjects have been previously reported in brief (Fratter *et al.*, 2011). The mean whole-group age at onset was 40 years (age range birth–70 years); patients with autosomal-recessive PEO presented considerably earlier at 7 years (age range birth–14 years), whereas those with autosomal-dominant PEO presented later in the fifth decade of life (mean age of onset 46 years; age range 15–70 years).

**Table 2:** Clinical, muscle histopathological and molecular genetics findings of 26 adult patients with *RRM2B* mutations

Patient	Clinical features	Age at onset (years)	Current age (years)	Affected relatives	Skeletal muscle histochemistry	Multiple mtDNA deletions	RRM2B cDNA change	Amino acid change	Exon	Previously published
Group A: Autosomal-dominant mutations										
1	Mild PEO, ptosis, proximal muscle weakness, ataxia and SNHL	63	64	Sister	n.d.	n.d.	c.48G>A	p.Glu16Glu	1	No
1.1	Mild PEO, ptosis, proximal muscle weakness, ataxia, hypothyroidism and SNHL	70	72	Sister of Patient 1	5% COX deficient/2% RRF	LRPCR	c.48G>A	p.Glu16Glu	1	No
2	PEO, ptosis, headaches, tinnitus, stroke-like episodes, fatigue and renal disturbance (renal calculi)	51	66	None	COX deficient/RRF	LRPCR, Southern blot	c.48G>A	p.Glu16Glu	1	No
3	Severe PEO, encephalopathy, stroke-like episodes, SNHL, cognitive decline and hypertension	59	71	None	10% COX deficient/RRF	LRPCR, qPCR	c.122G>A	p.Arg41Gln	2	Yes
4	PEO, ptosis, migraine, neck flexion weakness (mild) and proximal muscle weakness	60	Died age 66	None	8% COX deficient/1% RRF	LRPCR	c.121C>T	p.Arg41Trp	2	No
5	PEO, ptosis, ataxia, proximal muscle weakness, bulbar symptoms, facial weakness, IHD and sleep disorder	38	64	Sister	8% COX deficient/3% RRF	LRPCR, qPCR	c.208G>A	p.Asp70Asn	3	No
6	Severe PEO, ptosis, SNHL, dysphonia, proximal muscle weakness, ataxia, cataracts, glaucoma and IHD	50	75	Son	COX deficient/RRF	LRPCR	c.583G>A	p.Gly195Arg	6	Yes
6.1	PEO, ptosis	40	48	Son of Patient 6	n.d.	n.d. (father, 6, LRPCR)	c.583G>A	p.Gly195Arg	6	No
7	PEO, ptosis	30s	82	None	COX deficient/no RRF	LRPCR	c.632G>A	p.Arg211Lys	6	No
8	Severe PEO, diplopia, proximal muscle weakness, cataracts and migraine	48	62	None	COX deficient/no RRF	LRPCR	c.671T>G	p.Ile224Ser	6	No
9	Severe PEO, ptosis, mild dysphagia, fatigue, ataxia, glaucoma, bradycardia and hypertension	53	73	Father, brother paternal nephew	20% COX deficient/RRF	LRPCR, qPCR, Southern blot	c.950delT	p.Leu317X	9	Yes
10	Severe PEO, ptosis, proximal muscle weakness and ataxia	46	66	Mother	6% COX deficient/RRF	LRPCR, qPCR	c.950delT	p.Leu317X	9	Yes
11	PEO, ptosis	30	53	Mother	COX deficient/RRF plus non-specific myopathic and necrotic fibres	LRPCR, Southern blot	c.950delT	p.Leu317X	9	Yes
12	PEO, ptosis, TIAs, hypertension, dysarthria, dysphagia, fatigue and oral carcinoma	53	65	Mother	COX deficient/RRF	LRPCR, Southern blot	c.952G>T	p.Glu318X	9	Yes
13	PEO, DM, GI disturbance and fatigue	30	63	Brother, father, grandfather	COX deficient/RRF	LRPCR, Southern blot	c.965dupA	p.Asn322LysfsX4	9	Yes
14	Severe PEO, ptosis, mild dysphagia, fatigue, proximal muscle weakness, dysphonia, headaches, bradycardia, hypothyroidism, IBS and mild diastolic cardiac dysfunction	26	61	Maternal grandmother and aunt	3% COX deficient/RRF	LRPCR, qPCR Southern blot	c.965dupA	p.Asn322LysfsX4	9	Yes
15	PEO, ptosis, dysphagia and fatigue	54	64	Mother, sister	n.d.	n.d.	c.965dupA	p.Asn322LysfsX4	9	Yes
15.1	Severe PEO, ptosis, facial weakness, fatigue and respiratory failure	60 s	74	Sister of Patient 15	COX deficient/RRF	LRPCR	c.965dupA	p.Asn322LysfsX4	9	No
16	PEO, ptosis, ataxia and dysphagia	40 s	64	Mother	COX deficient/no RRF	LRPCR, Southern blot	c.965dupA	p.Asn322LysfsX4	9	No
17	Severe PEO, ptosis, diplopia, proximal muscle weakness, ataxia and fatigue	55	71	Daughter	n.d.	n.d.	c.979C>T	p.Arg327X	9	No
17.1	PEO, asymmetrical ptosis, ataxia and fatigue	43	48	Daughter of Patient 17	n.d.	n.d.	c.979C>T	p.Arg327X	9	No
18	PEO, ptosis, proximal muscle weakness, fatigue, tremor, impaired memory and breast carcinoma	45	62	Daughter	COX deficient/no RRF plus myopathic changes	LRPCR, Southern blot	c.1046C>G	p.Ala349Gly	9	No
Group B: Autosomal-recessive mutations										
19	Severe PEO, ptosis, proximal muscle weakness, facial weakness, scapulae winging, low BMI, hypogonadism and osteoporosis	14	37	None	>40% COX deficient/RRF	LRPCR, qPCR	c.431 C>T and c.817G>A	p.Thr144Ile and p.Gly273Ser	4 and 8	No
20	Severe PEO, asymmetrical ptosis, proximal and distal muscle weakness, ataxia, SNHL, facial weakness, low BMI, leukoencephalopathy and depression	11	43	None	30% COX deficient/5% RRF	LRPCR	c.556A>G and c.653C>T	p.Arg186Gly and p.Thr218Ile	6 and 6	No
21	PEO, ptosis, facial weakness, tongue weakness, neck weakness, SNHL, dysphagia, proximal muscle weakness, bilateral foot drop, renal failure (glomerulonephritis), DD, hypoparathyroidism and hypogonadism	Birth	Died age 25	None	COX deficient/RRF plus non-specific myopathy and increased lipid	LRPCR, Southern blot	c.431C>T and c.632G>A	p.Thr144Ile and p.Arg211Lys	4 and 6	Yes
22	PEO, ptosis, proximal muscle weakness, SNHL, fatigue and LD	4	15	None	COX deficient/RRF	LRPCR	c.606T>A and c.817G>A	p.Phe202Leu and p.Gly273Ser	6 and 8	Yes

RRF = ragged-red fibre; LRPCR = long-range PCR; qPCR = real-time PCR; SNHL = sensorineural hearing loss; TIA = transient ischaemic attack; BMI = body mass index; IBS = irritable bowel syndrome; IHD = ischaemic heart disease; DD = developmental delay; LD = learning difficulties; GI = gastrointestinal; n.d. = not determined; DM = diabetes mellitus; cDNA = complementary DNA; mtDNA = mitochondrial DNA; PEO = progressive external ophthalmoplegia.

#### Additional published adult cases

A systematic review of the literature to date (June 1, 2012) revealed published data on 18 *RRM2B-*positive adult families (Supplementary Table 1). Thirty-three individuals have been extensively genotyped and harbour *RRM2B* gene mutations characterized by either autosomal-recessive mitochondrial DNA depletion syndrome (Shaibani *et al.*, 2009) or recessive and dominant mutations that cause the accumulation of multiple mitochondrial DNA deletions (Tyynismaa *et al.*, 2009; Fratter *et al.*, 2011; Pitceathly *et al.*, 2011; Takata *et al.*, 2011).

### Major clinical features

Evaluation of the clinical features of all 31 adult patients harbouring pathogenic *RRM2B* mutations showed that PEO was universal and frequently associated with ptosis (28 patients). Neuromuscular features were common and included proximal muscle weakness (16 patients), bulbar dysfunction (13 patients) and fatigue (11 patients). Additional neurological manifestations included ataxia (12 patients) and sensorineural hearing loss (11 patients). Non-neurological sequelae such as gastrointestinal disturbance, including irritable bowel syndrome-like symptoms and low body mass index (six patients), and endocrinopathy, specifically hypothyroidism, hypoparathyroidism, diabetes and hypogonadism (seven patients), were also important clinical findings. Malignancy was detected in two patients (Patients 12 and 18).

Other less common problems included cognitive impairment (four patients); cardiac dysfunction, including non-fatal arrhythmia and diastolic dysfunction (two patients); encephalopathy and stroke-like events (three patients); cataracts (two patients); migraine (two patients); renal disturbance (three patients); distal muscle weakness (one patient); neuropathy (three patients); pigmentary retinopathy (two patients); short stature (one patient); glaucoma (one patient) and depression (three patients).

### Mitochondrial histochemical studies

**Figure 1:**
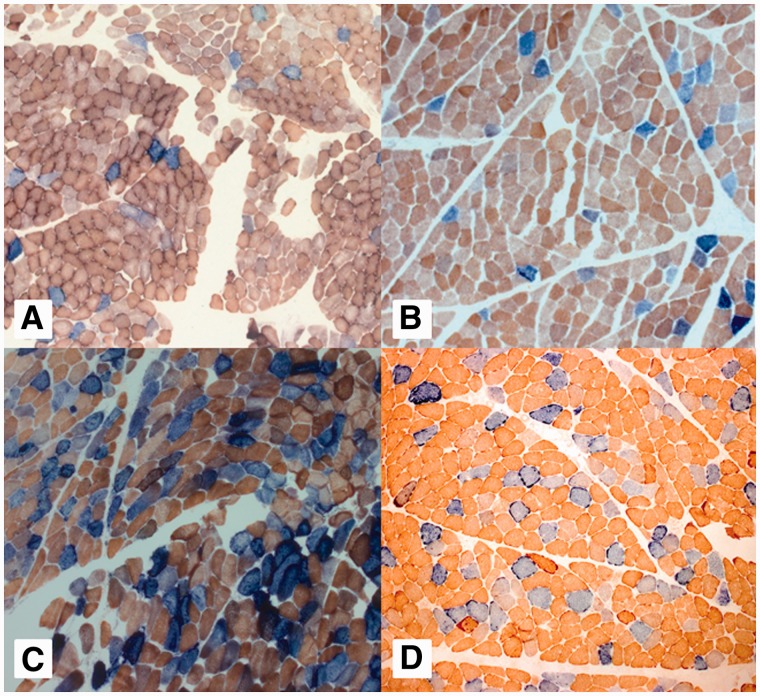
Mitochondrial histochemical changes associated with *RRM2B* mutations. Representative sequential COX-SDH histochemistry demonstrates a mosaic distribution of COX-deficient muscle fibres (blue) among fibres exhibiting normal COX activity (brown). Illustrated are the images for (**A**) Patient 5, (**B**) Patient 10, (**C**) Patient 19 and (**D**) Patient 20. Patients 5 and 10 have autosomal-dominant *RRM2B* mutations and a milder histochemical COX defect compared with Patients 19 and 20 (autosomal-recessive *RRM2B* mutations), in whom a more severe biochemical defect is clearly apparent.

Twenty-one patients with confirmed *RRM2B* mutations underwent a diagnostic muscle biopsy for the investigation of suspected mitochondrial disease. Patient 6.1 was diagnosed on the basis of *RRM2B* gene screening of a blood-derived DNA sample without performing a muscle biopsy. All showed histochemical evidence of a COX mosaic defect, with many exhibiting evidence of subsarcolemmal mitochondrial accumulation (ragged-red or ragged-blue fibres) after Gomori trichrome staining or SDH enzyme histochemistry. The severity of the associated COX mosaic defect varied widely, with a more pronounced histochemical defect associated with autosomal-recessive *RRM2B* mutations than autosomal-dominant mutations (Fig. 1).

### Mitochondrial DNA deletion analysis

**Figure 2:**
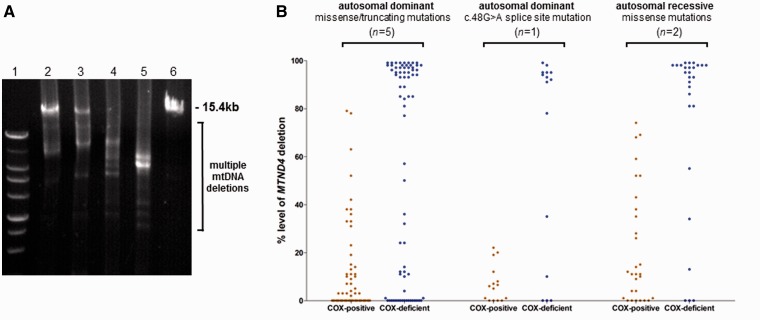
Characterization of multiple mitochondrial DNA deletions in muscle from patients with *RRM2B* mutations. (**A**) Representative long-range PCR amplification (15.4-kb fragment) across the major mitochondrial DNA arc shows evidence of multiple mitochondrial DNA deletions in patient muscle. Lane 1, size marker; Lane 2, Patient 5; Lane 3, Patient 10; Lane 4, Patient 19; Lane 5, Patient 20; Lane 6, Control subject. Patients with autosomal-dominant mutations (Lanes 2 and 3) show amplification of wild-type full-length mitochondrial DNA amplimers in addition to mitochondrial DNA deletions, whereas those with recessive *RRM2B* mutations (Patients 19 and 20) display a more severe secondary mitochondrial DNA defect. (**B**) Quantitative single-fibre real-time-PCR reveals the majority, but not all, of COX-deficient fibres contain high levels of a clonally expanded mitochondrial DNA deletion involving the *MT-ND4* gene. Autosomal- dominant missense or truncating mutations are represented by Patients 3, 5, 9, 10 and 14. Patient 1.1 has a dominant splicing mutation, whereas Patients 19 and 20 harbour recessive *RRM2B* mutations.

All 21 patients who underwent muscle biopsy had multiple mitochondrial DNA deletions detectable either by long-range PCR assays across the major mitochondrial DNA arc (Fig. 2) or by Southern blot analysis (undertaken in 11 of the 21 patients, with positive findings reported in all). A small number of patients (five patients with autosomal-dominant missense or truncating *RRM2B* mutations, one patient with an autosomal-dominant splicing defect and two patients with autosomal-recessive *RRM2B* mutations) were also investigated by real-time PCR to further characterize the presence of clonally expanded mitochondrial DNA deletions within single muscle fibres (Fig. 2). In all cases, the majority of COX-deficient fibres revealed very high levels (>80% mutated mitochondrial DNA) of clonally expanded mitochondrial DNA deletion involving the *MT-ND4* gene region (He *et al.*, 2002), whereas all COX-positive reacting fibres had lower levels of mitochondrial DNA deletion. No difference in the distribution of mitochondrial DNA deletion levels was observed between different *RRM2B* mutation types.

### Identification of pathogenic *RRM2B* mutations

**Figure 3:**
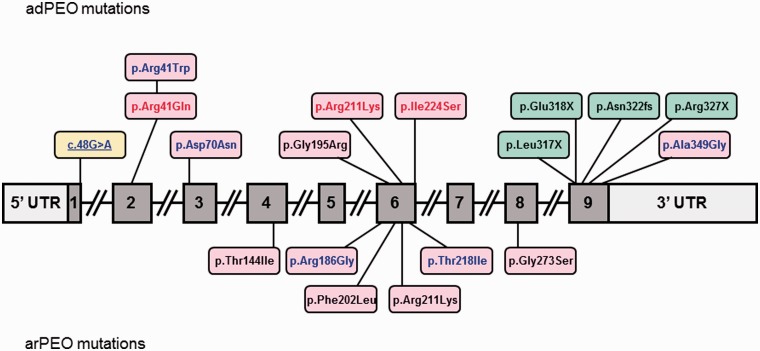
Schematic representation of the *RRM2B* gene structure illustrating the 18 different mutations identified in this study. Coding exons are numbered 1–9. Missense mutations are shown in pink boxes, exon 9 truncating mutations are shown in green boxes and the c.48G>A splice mutation is shown in a yellow box. *RRM2B* mutations associated with autosomal-dominant PEO (adPEO) in this study, which have also been associated with more severe autosomal-recessive disease [either early-onset autosomal-recessive PEO (arPEO) or mitochondrial DNA depletion syndrome], are highlighted in red. Novel unreported *RRM2B* mutations are highlighted in blue.

We identified 18 (12 dominantly and six recessively inherited) different *RRM2B* mutations within our cohort of patients, including five novel mutations that have not previously been reported (Fig. 3). Thirteen mutations were missense variants predicted to alter conserved amino acids (Supplementary Fig. 1), four were truncating mutations in exon 9 and one was a novel c.48G>A variant predicted to cause aberrant splicing.

Eleven patients (from nine families) were found to have truncating mutations in exon 9 of *RRM2B*, three with the p.Leu317X mutation, one with a p.Glu318X mutation, five with a p.Asn322LysfsX4 frameshift mutation and two patients from one family (Patient 17 and her clinically affected daughter, Patient 17.1) with the p.Arg327X mutation, which was the first mutation in this gene to be described as a cause of autosomal-dominant PEO (Tyynismaa *et al.*, 2009). All nine probands had affected family members and a family history consistent with a dominantly inherited disorder (Table 2). Eight patients (from seven families) harboured single heterozygous *RRM2B* missense changes, implying autosomal-dominant inheritance. In all seven families, multiplex ligation-dependent probe amplification was used to exclude exonic copy number variation *in trans*, with further supporting evidence of pathogenicity provided by the fact that these changes affected conserved residues (Supplementary Fig. 1). Four of these *RRM2B* mutations were unreported. Patient 4 harboured a novel p.Arg41Trp mutation, and although there was no relevant family history to support dominant transmission, mutation of this particular amino acid (p.Arg41Gln) was reported in another patient (Patient 3). Patient 5 had a novel p.Asp70Asn mutation and a clinically affected sister. Patient 18 had a novel mutation (p.Ala349Gly) in exon 9, which is a recognized mutation hotspot (Fratter *et al.*, 2011). The single heterozygous p.Ile224Ser mutation reported in Patient 8 is of particular interest, given that it has previously been reported as a recessive *RRM2B* mutation in a case of mitochondrial DNA depletion myopathy (Bornstein *et al.*, 2008) and, as such, seems to be able to behave as either a recessive or dominant allele. Similarly, p.Arg41Gln and p.Arg211Lys function as dominant alleles in Patients 3 and 7, respectively, but have also been identified in compound heterozygotes with more severe recessively inherited disease (Pitceathly *et al.*, 2011; and Patient 21). Four patients harboured two recessively inherited compound heterozygous *RRM2B* mutations associated with an earlier onset of disease and more pronounced COX histochemical defects (Fig. 1 and Table 2). The p.Thr144Ile and p.Gly273Ser mutations were reported *in trans* in two of the four patients, whereas the p.Arg186Gly and p.Thr218Ile mutations (Patient 20) were both novel. Within this family, the p.Thr218Ile mutation was detected in the heterozygous state in the clinically unaffected mother and a sister, whereas the second heterozygous mutation, p.Arg186Gly, was identified in another clinically unaffected sister. Segregation studies were also performed in the family of Patient 19; the p.Gly273Ser mutation was seen in the heterozygous state in the clinically unaffected mother, whereas a clinically unaffected sister was heterozygous for the p.Thr144Ile mutation alone. Paternal testing was not performed.

**Figure 4:**
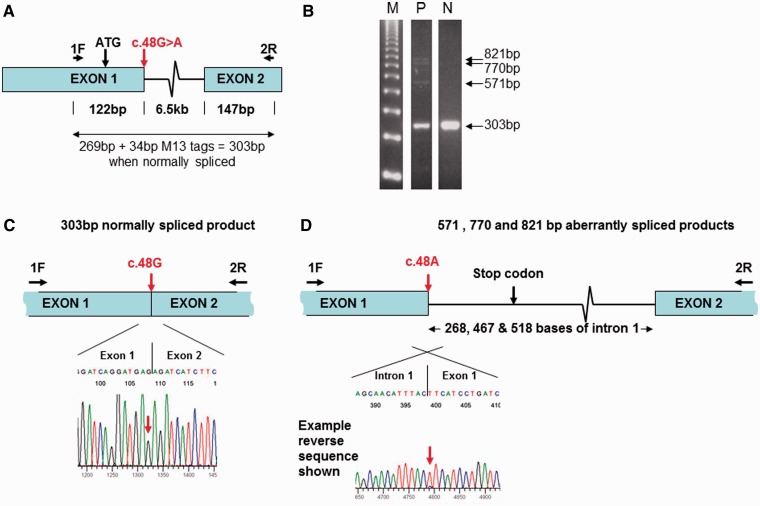
Molecular analysis of the novel c.48G>A (p.Glu16Glu) *RRM2B* mutation. (**A**) Schematic showing the location of primers and the c.48G>A mutation within *RRM2B* exons 1 and 2. (**B)** Agarose gel electrophoresis of amplified complementary DNA from Patient 1 (P) and a normal age-matched control (N) alongside a 123-bp molecular weight marker (M), with the position of normally spliced (303 bp) and aberrantly spliced (571, 770 and 821 bp) products indicated. (**C**) Sequencing of the normally spliced complementary DNA product from Patient 2 reveals that this product is exclusively derived from the normal c.48G allele (equivalent results for Patients 1 and 1.1 not shown). (**D**) Example of a sequencing trace of the aberrantly spliced products (571-bp fragment from Patient 2 is shown), demonstrating that these are almost exclusively derived from the mutant c.48G>A allele.

Finally, an unreported c.48G>A *RRM2B* variant was identified in three patients (Patients 1, 1.1 and 2). This is not predicted to alter the amino acid sequence, resulting in a predicted p.Glu16Glu synonymous change. However, it was noted that the c.48G>A variant occurs at the last nucleotide of exon 1 and was therefore considered likely to result in aberrant splicing (Fig. 4). Analysis of blood RNA extracted from Patients 1, 1.1 and 2 across the exon 1–2 junction demonstrated that normally spliced RNA arises exclusively from the normal c.48G allele (Fig. 4). Furthermore, the three patients had additional RNA species associated with the c.48G>A allele, in which various portions of the 5′ region of intron 1 were retained, resulting in the introduction of a termination codon, 26 codons beyond exon 1. All three patients had RNA incorporating the first 268 nucleotides of intron 1. Patient 1.1 also had RNA with the first 518 nucleotides of intron 1, and Patient 1 also had RNA with the first 467 and 518 nucleotides of intron 1 (Fig. 4). Thus, these results demonstrate that c.48G>A abolishes normal splicing of exons 1 to 2, leading to partial intron retention and premature termination of translation, thereby confirming pathogenicity of c.48G>A.

## Discussion

*RRM2B* mutations are emerging as one of the leading causes of both paediatric and adult-onset mitochondrial disease associated with disruption of mitochondrial DNA maintenance. *RRM2B* mutations represent the third most common cause of Mendelian PEO and multiple mitochondrial DNA deletions in adults (13%), following mutations in *POLG* (27%) and *PEO1* (14%), based on data from both Oxford and Newcastle centres. It is clear there is significant clinical overlap between the multiple mitochondrial maintenance genes. In our cohort, ophthalmoparesis was universal, usually severe and often associated with ptosis (90% of patients). Other prominent myopathic features included proximal muscle weakness (52% of patients) and bulbar dysfunction (42% of patients). The latter manifested as dysarthria, dysphagia, dysphonia, facial weakness and neck weakness. Sensorineural hearing loss (36% of patients) and gastrointestinal disturbance (19% of patients), including irritable bowel syndrome-like symptoms and low body mass index, were also relatively common findings. This is perhaps unsurprising, given that they are prominent features in children with mitochondrial DNA depletion secondary to *RRM2B* mutations. The presence of significant bulbar weakness, hearing loss and gastrointestinal symptoms should guide clinicians towards *RRM2B* genetic analysis before *POLG* and *PEO1*, given that these key features are more common components of the clinical spectrum (bulbar dysfunction 42% versus 37% and 12%; sensorineural hearing loss 36% versus 11% and 9%; and gastrointestinal symptoms 19% versus unreported and 9% in *RRM2B* versus *POLG* and *PEO1*-related PEO syndromes, respectively) (Horvath *et al.*, 2006; Fratter *et al.*, 2011).

Although cerebellar ataxia was present in 39% of cases, other central features of mitochondrial disease were seen less frequently, such as cognitive impairment (13% of patients) and encephalopathy/stroke-like events (10% of patients). Cardiac complications were rare (10%) and non-fatal. Disturbance in renal function was present in three patients, resulting from obstructive uropathy (two patients) and glomerulonephritis (one patient), with no reports of proximal renal tubulopathy, a common finding in children with *RRM2B* mutations and mitochondrial DNA depletion.

Two patients developed solid tumours (Patient 12 had oral carcinoma, and Patient 18 had breast carcinoma). *RRM2B* is a gene not only involved in mitochondrial DNA replication but also plays a critical role in DNA damage repair. p53R2, a p53-inducible homologue of the R2 subunit of ribonucleotide reductase, has been evaluated in different cancer types and is known to play a critical role in DNA damage repair and cancer cell proliferation (Zhang *et al.*, 2011). These data may suggest abnormalities in DNA repair; however, it is not possible to conclude whether *RRM2B* mutations are oncogenic, owing to small sample size and lack of mutational analysis of tumour tissue.

**Figure 5:**
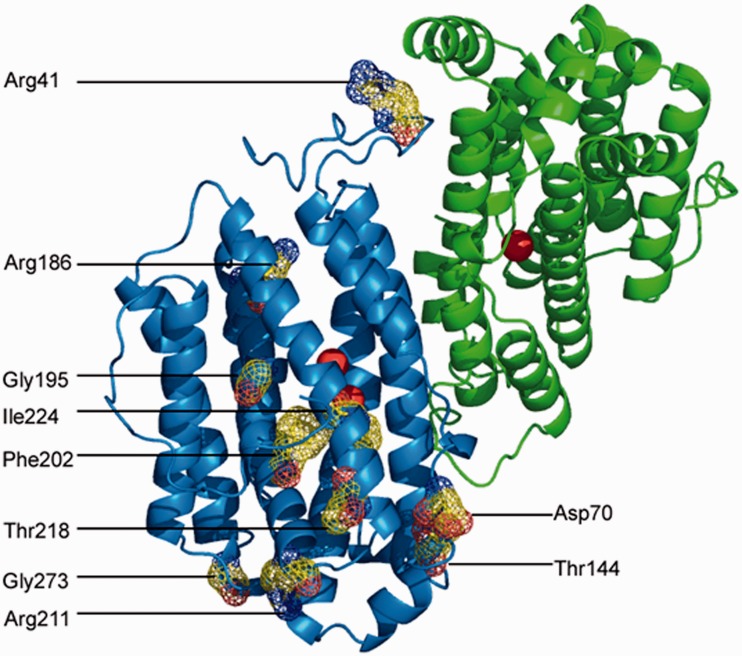
Location of missense *RRM2B* mutations on crystal structure. An image of the p53R2 dimer structure is shown (Protein Data Bank code 3HF1). The locations of 11 of the 12 amino acids altered by missense mutations identified in this study are shown on the di-iron bound (active) subunit (it has not been possible to illustrate Ala349, as the C-terminus is absent from the crystal structure). The physical space occupied by these 11 amino acids is illustrated by a multicoloured mesh, with blue indicating positive charge, red indicating negative charge and yellow indicating neutral. Iron atoms are represented as red spheres.

To further understand the functional consequence of the *RRM2B* mutations identified, we mapped the positions of the mutated amino acids on the tertiary p53R2 structure (Smith *et al.*, 2009) (Fig. 5). Many of the missense mutations identified appear likely to affect the iron-binding properties of p53R2, and hence impair the catalytic capability of the functional heterotetramer (two p53R2 subunits and two R1 subunits). Gly195, Phe202 and Ile224 are located around the iron-binding pocket. Although the effect of Phe202Leu may be orchestrated through subtle hydrophobic contacts, the effect of amino acids Gly195 and Ile224 is more blatant. Positioned adjacent to amino acids that contribute to the iron coordination environment, substitutions at these locations (p.Gly195Arg and p.Ile224Ser) will influence their amino acid neighbours and alter the coordination of the iron atom(s). Previous molecular modelling has indicated that p.Arg41Gln prevents formation of a salt bridge that is important in conformational changes that control iron binding (Smith *et al.*, 2009; Pitceathly *et al.*, 2011). p.Arg41Trp is also predicted to prevent formation of this salt bridge. Arg211 forms a salt bridge to Glu85, which is thought to be important in stabilization of the di-iron form (Smith *et al.*, 2009; Pitceathly *et al.*, 2011), and therefore p.Arg211Lys may also destabilize the di-iron subunit.

Thr144, Arg186, Thr218 and Gly273 are all located at the end of, or between in the case of Gly273, α-helices and appear to stabilize the orientation of the helices. Mutation of these four amino acids may reduce protein folding efficiency and is associated with autosomal-recessive disease in our cohort. The effect of the p.Asp70Asn mutation cannot readily be predicted, as Asp70 lies in a poorly understood region of the protein between two helices. p.Ala349Gly could not be modelled because the crystal structure does not include the C-terminal portion of the protein. However, Ala349 is located within a conserved heptapeptide (amino acids 345–351) required for interaction with the R1 subunit (Tyynismaa *et al.*, 2009), and loss of this heptapeptide has been proposed as the pathological basis of the exon 9 truncating mutations (Tyynismaa *et al.*, 2009).

There was a clear relationship between phenotypic severity and genotype in the patients studied. Individuals harbouring recessively inherited compound heterozygous *RRM2B* mutations (Patients 19, 20, 21 and 22) presented at an earlier age (mean age of onset 7 years) with a more severe and multisystem disorder, whereas patients with single heterozygous mutations, inferring autosomal-dominant transmission, had a later average age of disease onset (46 years), as is seen with *PEO1* mutations (Horvath *et al.*, 2006), and developed a predominantly myopathic phenotype consisting of PEO, ptosis, proximal muscle weakness and bulbar dysfunction, with exceptions noted (Patients 3 and 14). The distinction between recessively and dominantly inherited mutations was also evident on histochemical analysis of skeletal muscle tissue, in which COX-deficient fibres were much more widespread in patients with recessively inherited compound heterozygous mutations (Fig. 1).

Clinical syndromes caused by dysfunction of the nuclear maintenance genes can be broadly classified into two groups: (i) mutations that cause mitochondrial DNA depletion, which are at the most severe end of the phenotypic spectrum; and (ii) mutations that predispose to accumulation of multiple mitochondrial DNA deletions. The latter is further subdivided into: (a) recessively inherited disease, which presents during childhood with multisystem involvement; and (b) dominantly inherited disease, which is milder, typically develops in adulthood, and is often tissue-specific. A review of the literature suggests *RRM2B* generally conforms to these basic principles. The most severe form of *RRM2B*-related mitochondrial disease is associated with mitochondrial DNA depletion (Bourdon *et al.*, 2007; Bornstein *et al.*, 2008; Acham-Roschitz *et al.*, 2009; Kolberg *et al.*, 2009; Spinazzola *et al.*, 2009). Clinical presentation occurs in the first 6 months of life, with a multisystem disorder characterized by muscle hypotonia and weakness, seizures, gastrointestinal dysmotility, respiratory insufficiency, hearing loss, lactic acidosis, renal tubulopathy and early childhood mortality. The first reported adult-onset case was in a 30-year-old female with mitochondrial neurogastrointestinal encephalopathy who harboured compound heterozygous missense mutations in *RRM2B* and mitochondrial DNA depletion (Shaibani *et al.*, 2009). *RRM2B* mutations were subsequently associated with multiple mitochondrial DNA deletions in two large unrelated families with autosomal-dominant PEO and an identical heterozygous nonsense mutation that caused truncation of the translated p53R2 protein (Tyynismaa *et al.*, 2009). Further reports have demonstrated *RRM2B* mutations with Kearns–Sayre syndrome and sporadic/familial PEO (Fratter *et al.*, 2011; Pitceathly *et al.*, 2011).

There are, however, notable exceptions to these rules. First, *RRM2B*-related mitochondrial DNA depletion can potentially cause a relatively mild clinical phenotype (Shaibani *et al.*, 2009); and second, identical *RRM2B* mutations are associated with a varied phenotypic severity, depending on whether they exist in homozygous, compound heterozygous or heterozygous states. We attempt to explain the latter finding using molecular modelling of the *RRM2B* missense mutations identified in this study, which suggests the variants can be broadly divided into two groups: mutations that severely impair ribonucleotide reductase activity and cause autosomal-dominant disease through a dominant-negative effect; and mutations associated with autosomal-recessive disease, which are predicted to result in moderately decreased catalytic activity or decreased levels of functional protein through reduced protein folding efficiency. This is likely to be a consequence of the heterotetrameric structure of ribonucleotide reductase, which predisposes the enzyme to both a dominant-negative effect (competitive binding and inactivation of the enzyme) or a gain-of-function effect (competitive binding with altered function of the enzyme), alongside the loss of enzymatic activity that occurs with recessively inherited disease. There is also evidence that impaired assembly of the multiprotein structures occurs with some mutations, and there may be a dosage effect, whereby wild-type p53R2 appears to partially compensate for the mutant allele, thus ameliorating the clinical phenotype when present in a heterozygous state (Pitceathly *et al.*, 2011). This phenomenon is demonstrated in the present study by the p.Ile224Ser, c.48G>A, p.Arg41Gln and p.Arg211Lys variants. These four variants are associated with relatively late-onset PEO (fourth to eighth decades) and multiple mitochondrial DNA deletions when present as the only heterozygous change, as in Patients 1, 1.1, 2, 3, 7 and 8. However, when homozygous or compound heterozygous, these variants are associated with much more severe multisystem disease, either with mitochondrial DNA depletion in the case of p.Ile224Ser (homozygote reported by Bornstein *et al*., 2008) and c.48G>A (compound heterozygous with another pathogenic *RRM2B* mutation, Oxford Molecular Genetics Laboratory, unpublished data), or with multiple mitochondrial DNA deletions in the case of p.Arg41Gln and p.Arg211Lys (compound heterozygous patient reported by Pitceathly *et al.*, 2009, and Patient 21 in this study, respectively).

Our data support previous reports that *RRM2B* mutations can present with a PEO-plus/Kearns–Sayre syndrome phenotype akin to single mitochondrial DNA deletion disorders (Pitceathly *et al.*, 2011). Patients 19, 20, 21 and 22 all developed symptoms before the age of 20 years, with PEO and other features suggestive of Kearns–Sayre syndrome, such as hearing loss, cerebellar ataxia and endocrine disturbance, although pigmentary retinopathy and heart block were absent. We would, therefore, recommend *RRM2B* over *POLG* and *PEO1* genetic analysis in patients with PEO-plus or Kearns–Sayre syndrome before a muscle biopsy is performed to exclude a single mitochondrial DNA deletion, if there is a Mendelian pattern of inheritance. *RRM2B* analysis should also be considered in patients with mitochondrial neurogastrointestinal encephalopathy if blood/urinary deoxyuridine and thymidine levels are undetectable and thymidine phosphorylase activity is normal in white cells and platelets, based on the previous report of mitochondrial neurogastrointestinal encephalopathy secondary to compound heterozygous missense mutations in *RRM2B* (Shaibani *et al.*, 2009), Case 20 reported here and because gastrointestinal symptoms were a prominent finding in our cohort of adult patients.

Finally, we report the novel c.48G>A *RRM2B* variant and provide evidence to support its pathogenicity through aberrant splicing, partial intron retention and premature termination of translation and hence predicted absence of any functional protein from this allele. We report two families (Patients 1, 1.1 and 2) where heterozygosity for c.48G>A is associated with adult-onset mitochondrial disease. Because all other *RRM2B* mutations reported to date in this disease group are either missense or exon 9 truncating, our data further expand the molecular heterogeneity of *RRM2B*-related adult mitochondrial disease.

## Conclusion

Prioritizing which nuclear-encoded mitochondrial maintenance genes to screen in adults with multiple mitochondrial DNA deletions is challenging. These data provide the physician with important clinical information regarding the phenotypic spectrum of *RRM2B*-related adult mitochondrial disease and should help guide genetic diagnosis and enable tailored counselling regarding potential disease progression. PEO, ptosis and proximal muscle weakness are well recognized as the predominant clinical features seen in adult patients with *POLG* and *PEO1* (*C10ORF2*; Twinkle) mutations. Bulbar dysfunction, hearing loss and gastrointestinal problems, including irritable bowel syndrome-like symptoms and low body mass index, are also additional discriminatory features seen in *RRM2B*-related mitochondrial disease and appear to occur more often than with the other individual nuclear-encoded mitochondrial maintenance genes. CNS involvement, characteristic of other syndromic presentations of mitochondrial disease, is present less frequently. Thus, the prominence of bulbar dysfunction, gastrointestinal problems and hearing loss, in the absence of conspicuous CNS features, would support early prioritization of screening of *RRM2B* over *POLG* and *PEO1* in adults with PEO and muscle-restricted multiple mitochondrial DNA deletions. Furthermore, *RRM2B* gene analysis should be considered early in Kearns–Sayre syndrome where there is evidence of multiple mitochondrial DNA deletions in skeletal muscle or when there appears to be a Mendelian pattern of inheritance. Finally, *RRM2B* screening should be considered in patients with mitochondrial neurogastrointestinal encephalopathy when deoxyuridine and thymidine levels in both blood and urine are negative, thymidine phosphorylase activity is normal in white cells and platelets and analysis of the *TYMP* gene does not identify causative mutations.

## Supplementary material

Supplementary material is available at *Brain* online.

## Funding

This work was funded by the following grants: MRC Centre for Translational Research in Neuromuscular Disease Mitochondrial Disease Patient Cohort (UK) (G0800674); Wellcome Trust Centre for Mitochondrial Research (906919); MRC Centre for Neuromuscular Diseases (G0601943); Newcastle University Centre for Brain Ageing and Vitality supported by BBSRC, EPSRC, ESRC and MRC (G0700718); Wellcome Trust Programme Grant (074454/Z/04/Z); UK NIHR Biomedical Research Centre for Ageing and Age-related disease award to the Newcastle upon Tyne Foundation Hospitals NHS Trust. M. D. is a member of the German network mitoNET funded by the German Federal Ministry of Education and Research (BMBF).

## Supplementary Material

Supplementary Data

## Abbreviations

COX: cytochrome *c* oxidase
dNTP: deoxyribonucleoside triphosphate
PEO: progressive external ophthalmoplegia
SDH: succinate dehydrogenase
