# Supplementary material for: Adults with RRM2B-related mitochondrial disease have distinct clinical and molecular characteristics
Source: Brain. 2012 Oct 29;135(11):3392–403. doi: 10.1093/brain/aws231 (PMC3501970; doi:10.1093/brain/aws231)
Supplement: Supplementary Data [file supp_135_11_3392__index.html]

Adults with RRM2B-related mitochondrial disease have distinct clinical and molecular characteristics — Supplementary Data 

# Adults with *RRM2B*-related mitochondrial disease have distinct clinical and molecular characteristics

## Supplementary Data

files

**Files in this Data Supplement:**

- Supplementary Data - doc file
- Supplementary Data - doc file
- Supplementary Data - docx file
