# Supplementary material for: Adults with RRM2B-related mitochondrial disease have distinct clinical and molecular characteristics
Source: Brain. 2012 Oct 29;135(11):3392–403. doi: 10.1093/brain/aws231 (PMC3501970; doi:10.1093/brain/aws231)
Supplement: Supplementary Data [file supp_aws231_E-supplemental_Figure_1_revised_RRM2B_Brain_2012.doc]

Human MGDPERP----EAAGL----------------------D-----QDERSS 19

Chimp MGDPERP----EAAGL----------------------D-----QDERSS

Macaque MGDPERP----EAAGL----------------------D-----LDERSS

Mouse MGDPERP----EAARP----------------------E-----KGEQLC

Dog MGDPERP----EAARP----------------------E-----PDERLS

Chicken --------------------------------------------------

Frog --------------------------------------------------

Pufferfish LVDKSPG----WG-------------------------------------

*C.elegans* MTLTEIQNVEKENAGASVPKHSSNKLKLEKELEKLEIVDQTKAASAEETN

*S.cerevisiae* MEAHNQ----F-----------------------------LKTF-----Q

Human SDTNESEIKSNEEPLLRKSSR**R**FVIFPIQYPDIWKMYKQAQASFWTAEEV 69

Chimp SDTNESEIKSNEEPLLRKSSR**R**FVIFPIQYPDIWKMYKQAQASFWTAEEV

Macaque SDTNDNEIKSNEEPLLRKSSR**R**FVIFPIQYPDIWKMYKQAQASFWTAEEV

Mouse SETEENVVRSNEEPLLRKSSR**R**FVIFPIQYPDIWRMYKQAQASFWTAEEV

Dog SDTNENEVKSDEEPLLRKSSR**R**FVIFPIQYPDIWKMYKQAQASFWTAEEV

Chicken ------GLKPHEEPLLRKNPR**R**FVIFPIQHPDIWKMYKQAQASFWTAEEV

Frog ----------EEEPFLRKNPQ**R**FVIFPIHYPDIWKMYKKAQASFWTAEEV

Pufferfish -----KGSGTEGEPLLQENPR**R**FVIFPIQYPDIWKMYKQAQASFWTVEEV

*C.elegans* NESEVNEL-DADEPMLQDLDN**R**FVIFPLKHHDIWNFYKKAVASFWTVEEV

*S.cerevisiae* KERHDMKEAEKDEILLMENSR**R**FVMFPIKYHEIWAAYKKVEASFWTAEEI

Human **D**LSKDLPHWNKLKADEKYFISHILAFFAASDGIVNENLVERFSQEVQVPE 119

Chimp **D**LSKDLPHWNKLKADEKYFISHILAFFAASDGIVNENLVERFSQEVQVPE

Macaque **D**LSKDLPHWNKLKADEKYFISHILAFFAASDGIVNENLVERFSQEVQVPE

Mouse **D**LSKDLPHWNKLKSDEKYFISHILAFFAASDGIVNENLVERFSQEVQVPE

Dog **D**LSKDLPHWNKLKSDEKYFISHILAFFAASDGIVNENLVERFSQEVQVPE

Chicken **D**LSKDLPHWNKLKADEKYFISHVLAFFAASDGIVNENLVARFSQEVQIPE

Frog **D**LSKDLVHWEKLKPEERNFISHILAFFAASDGIVNENLVERFSQEVQVPE

Pufferfish **D**LSKDLAHWDSLKPEEKHFISHVLAFFAASDGIVNENLVQRFCQEVQVPE

*C.elegans* **D**LGKDMNDWEKMNGDEQYFISRILAFFAASDGIVNENLCERFSNEVQVSE

*S.cerevisiae* ELAKDTEDFQKLTDDQKTYIGNLLALSISSDNLVNKYLIENFSAQLQNPE

Human ARCFYGFQILIENVHSEMYSLLID**T**YIRDPKKREFLFNAIETMPYVKKKA 169

Chimp ARCFYGFQILIENVHSEMYSLLID**T**YIRDPKKREFLFNAIETMPYVKKKA

Macaque ARCFYGFQILIENVHSEMYSLLID**T**YIRDPKKREFLFNAIETMPYVKKKA

Mouse ARCFYGFQILIENVHSEMYSLLID**T**YIRDPKKREFLFNAIETMPYVKKKA

Dog ARCFYGFQILIENVHSEMYSLLID**T**YIRDPKKREFLFNAIETMPYVKKKA

Chicken ARCFYGFQILIENVHSEMYSLLID**T**YIKDPEKRDFLFNAIETMPCVKKKA

Frog ARCFYGFQILIENVHSEMYSLLIE**T**YIKDPRRREFLFNAIETMPCVRKKA

Pufferfish ARSFYSYQVLIESVHSEMYSLLIN**T**YIRDLKEREHLFNAIYTMPCVKRKA

*C.elegans* ARFFYGFQIAIENIHSEMYSKLIE**T**YIRDETERNTLFNAVDEFEFIKKKA

*S.cerevisiae* GKSFYGFQIMMENIYSEVYSMMVDAFFKDPKNI-PLFKEIANLPEVKHKA

Human DWALRWIADRKSTFGE**R**VVAFAAVE**G**VFFSGS**F**AAIFWLKK**R**GLMPGL**T**F 219

Chimp DWALRWIADRKSTFGE**R**VVAFAAVE**G**VFFSGS**F**AAIFWLKK**R**GLMPGL**T**F

Macaque DWALRWIADRKSTFGE**R**VVAFAAVE**G**IFFSGS**F**AAIFWLKK**R**GLMPGL**T**F

Mouse DWALRWIADRKSTFGE**R**VVAFAAVE**G**IFFSGS**F**AAIFWLKK**R**GLMPGL**T**F

Dog DWALRWIEDRKSTFGE**R**VVAFAAVE**G**IFFSGS**F**AAIFWLKK**R**GLMPGL**T**F

Chicken DWALKWIEDRESTFGE**R**VVAFAAVE**G**IFFSGS**F**AAIFWLKK**R**GLMPGL**T**F

Frog QWALRWISDRKASFGE**R**VVAFAAVE**G**IFFSGS**F**AAIFWLKK**R**GLMPGL**T**F

Pufferfish DWALQWINDSTSTFGE**R**LVAFAAVE**G**IFFSGS**F**ASIYWLKK**R**GLMPGL**T**Y

*C.elegans* DWALRWISDKKASFAE**R**LIAFAAVE**G**IFFSGS**F**ASIFWLKK**R**GLMPGL**T**H

*S.cerevisiae* AFIERWISNDDSLYAE**R**LVAFAAKE**G**IFQAGNYASMFWLTDKKIMPGLAM

Human SNEL**I**SRDE**G**LHCDFACLMFQYLVNKPSEERVREIIVDAVKIEQEFLTEA 269

Chimp SNEL**I**SRDE**G**LHCDFACLMFQYLVNKPSEERVREIIVDAVKIEQEFLTEA

Macaque SNEL**I**SRDE**G**LHCDFACLMFQYLVNKPSEERVREIIVDAVQIEQEFLTEA

Mouse SNEL**I**SRDE**G**LHCDFACLMFQYLVNKPSEDRVREIIADAVQIEQEFLTEA

Dog SNEL**I**SRDE**G**LHCDFACLMFQYLVNKPSEERVREIIVNAVEIEQEFLTEA

Chicken SNEL**I**SRDE**G**LHCDFACLMFHYLVNRPSEERVREIIVNAVEIEQEFLTEA

Frog SNEL**I**SRDE**G**LHTDFACLMFHYLVKKPTEERVTEIIVNAVRIEQEFLTEA

Pufferfish SNEL**I**SRDE**G**LHCTFACLLYSYLVKKPSEDRVKDIITKAVSIEQEFLTEA

*C.elegans* SNEL**I**SRDE**G**LHRDFACLLYSKLQKKLTQQRIYDIIKDAVAIEQEFLTEA

*S.cerevisiae* ANRN**I**CRDR**G**AYTDFSCLLFAHLRTKPNPKIIEKIITEAVEIEKEYYSNS

Human LPV**G**LIGMNCILMKQYIEFVADRLLVELGFSKVFQAENPFDFMENIS**LE**G 319

Chimp LPV**G**LIGMNCILMKQYIEFVADRLLVELGFSKVFQAENPFDFMENISLEG

Macaque LPV**G**LIGMNCILMKQYIEFVADRLLVELGFSKIFQAENPFDFMENISLEG

Mouse LPV**G**LIGMNCVLMKQYIEFVADRLLGELGFSKIFQAENPFDFMENISLEG

Dog LPV**G**LIGMNCVLMKQYIEFVADRLLVELGFSKVFQAENPFDFMENISLEG

Chicken LPV**G**LIGMNCTLMKQYIEFVADRLLMELGFSKVFHAENPFDFMENISLEG

Frog LPVSLIGMNCTLMKTYIEFVADRLLVELGCSKAFKAENPFDFMENISLEG

Pufferfish LPVDLIGMNRCLMKQYIEFVADRLFADLGLAKMYNAENPFDFMEAISLEG

*C.elegans* LPVDMIGMNCRLMSQYIEFVADHLLVELGCDKLYKSKNPFDFMENISIDG

*S.cerevisiae* LPVEKFGMDLKSIHTYIEFVADGLLQGFGNEKYYNAVNPFEFMEDVATAG

Human KT**N**FFEK**R**VSEYQRFAVMAET--TDNVFTLD**A**DF 351

Chimp KTNFFEKRVSEYQRFAVMAET--TDNVFTLD**A**DF

Macaque KTNFFEKRVSEYQRFAVMAET--TDNVFTLD**A**DF

Mouse KTNFFEKRVSEYQRFAVMAET--TDNVFTLD**A**DF

Dog KTNFFEKRVAEYQRFAVMAET--TDNVFTLD**A**DF

Chicken KTNFFEKRVSEYQRFAVMAET--MDNVFTLD**A**DF

Frog KTNFFEKRVSEYQRFAVMAHT--EDNVFTLD**A**DF

Pufferfish KTNFFEKRVAEYQRFGIMSSP--VDSEFTLD**A**DF

*C.elegans* KTNFFEKRVSEYQRPGVMVNE--AERQFDLE**A**DF

*S.cerevisiae* KTTFFEKKVSDYQKASDMSKSATPSKEINFDDDF

**CLUSTAL W (1.83) multiple sequence alignment of *RRM2B* sequences.** The locations of amino acids altered by the 12 different missense substitutions (p.Arg41Gln, p.Arg41Trp, p.Asp70Asn, p.Thr144Ile, p.Arg186Gly, p.Gly195Arg, p.Phe202Leu, p.Arg211Lys, p.Thr218Ile, p.Ile224Ser, p.Gly273Ser & p.Ala439Gly) are highlighted in red. The locations of the 4 different truncating mutations (p.Leu317X, p.Glu318X, p.Asn322LysfsX4 & p.Arg327X) are highlighted in blue.
