# Supplementary material for: Adults with RRM2B-related mitochondrial disease have distinct clinical and molecular characteristics
Source: Brain. 2012 Oct 29;135(11):3392–403. doi: 10.1093/brain/aws231 (PMC3501970; doi:10.1093/brain/aws231)
Supplement: Supplementary Data [file supp_aws231_E-supplemental_Figure_1_revised_RRM2B_Brain_2012_2.doc]

**Supplemental Figure 1:** Evolutionary conservation of mutated amino acids detected in our *RRM2B* patient cohort.

**Supplemental Table 1:** A summary of the published clinical data for adults with *RRM2B* mutations
