# Supplementary material for: Adults with RRM2B-related mitochondrial disease have distinct clinical and molecular characteristics
Source: Brain. 2012 Oct 29;135(11):3392–403. doi: 10.1093/brain/aws231 (PMC3501970; doi:10.1093/brain/aws231)
Supplement: Supplementary Data [file supp_aws231_Supplementary_Table_1.docx]

| **Amino acid change** | **Nucleotide change** | **Genotype** | **Effect on mtDNA** | **Number of affected individuals / families** | **Clinical features (number of affected individuals in brackets)** | **Age of onset** | **Outcome** | **Reference** |
| --- | --- | --- | --- | --- | --- | --- | --- | --- |
| p.Arg110His  p.Arg121His | c.329G>A  c.362G>A | Compound heterozygous | Depletion | 1 / 1 | Ataxia  Cachexia  Dysarthria  Gastrointestinal dysmotility  Hearing loss  PEO  Peripheral neuropathy  Ptosis | 30 years | Alive 42 years | Shaibani *et al*. (2009) |
| p.Arg327X | c.979C>T | Heterozygous | Multiple deletions | 17 / 2 | Ataxia  Cognitive dysfunction  Exercise intolerance  Hypoacussis  Mood disturbance  PEO  Ptosis  Reduced reflexes | 3^rd^ decade (subject 3, family 2) | Not available | Tyynismaa *et al*. (2009) |
| p.Thr144Ile  p.Arg211Lys  p.Phe202Leu  p.Gly273Ser  p.Leu317X  p.Glu318X  p.Asn322LysfsX4  p.Arg41Gln  p.Gly195Arg  p.Gly229Val | c.431C>T  c.632G>A  c.606T>A  c.817G>A  c.950delT  c.952G>T  c.965dupA  c.122G>A  c.583G>A  c.686G>T | Compound heterozygous  Compound heterozygous  Heterozygous  Heterozygous  Heterozygous  Heterozygous  Heterozygous  Heterozygous | Multiple deletions  Multiple deletions  Multiple deletions  Multiple deletions  Multiple deletions  Multiple deletions  Multiple deletions  Multiple deletions | 1 / 1  1 / 1  3 / 3  1 / 1  3 / 3  1 / 1  1 / 1  1 / 1 | Areflexia (1)  Ataxia (1)  Cataracts (1)  Cognitive impairment (1)  Developmental delay (1)  Diabetes Mellitus (1)  Dysarthria (2)  Dysphagia (5)  Dysphonia (2)  Encephalopathy (1)  Facial weakness (1)  Fatigue (4)  Gastrointestinal disturbance (2)  Glaucoma (1)  Hearing loss (5)  Hypogonadism (1)  Hypoparathyroidism (1)  Myopathy (5)  PEO (8)  Ptosis (7)  Renal failure (1)  Stroke-like episodes (1) | Birth  4 years  30-53 years  53 years  26-54 years  59 years  50 years  15 years | Died 25 years  Alive 14 years  Alive 52-73 years  Alive 64 years  Alive 61-64 years  Alive 71 years  Alive 75 years  Alive 25 years | Fratter *et al*. (2011) |
| p.Arg41Gln  p.Glu131Lys | c.122G>A  c.391G>A | Compound heterozygous | Multiple deletions | 1 / 1 | Cachexia (1)  Delayed puberty (1)  Fatigue (2)  Hearing loss (1)  Hydronephrosis (1)  PEO (2)  Pigmentary retinopathy (1)  Proximal muscle weakness (1)  Ptosis (2)  Raised CK (1)  Raised CSF lactate (1)  Raised CSF protein (1)  Short stature (1) | 4 years | Died 22 years | Pitceathly *et al.* (2011) |
| p.Glu85del | c.253_255delGAG | Heterozygous | Multiple deletions | 1 / 1 |  | 58 years | Died 66 years |  |
| p.Pro33Ser | c.341G>A | Homozygous | Multiple deletions | 1 / 1 | Deafness  Depression  Gonadal atrophy  Muscle weakness  PEO  Pigmentary retinopathy  Ptosis | 16 years | Alive at 43 years | Takata *et al.* (2011) |

Abbreviations: PEO = progressive external ophthalmoplegia; CK = creatine kinase
